# Phenolic content and antioxidant activity of *Pereskia grandifolia* Haw. (Cactaceae) extracts

**DOI:** 10.4103/0973-1296.66945

**Published:** 2010

**Authors:** K. S. Sim, A. M. Sri Nurestri, A. W. Norhanom

**Affiliations:** *Institute of Biological Sciences, Faculty of Science, University of Malaya, 50603 Kuala Lumpur, Malaysia*; 1 *Centre for Foundation Studies in Science, University of Malaya, 50603 Kuala Lumpur, Malaysia*

**Keywords:** Antioxidant activity, Cactaceae, *Pereskia grandifolia*, phenolic content

## Abstract

The leaves of ***Pereskia grandifolia*** Haw. (Cactaceae), commonly known as “Jarum Tujuh Bilah” in Malaysia, have been traditionally used as natural remedy in folk medicine by the locals. In the present study, the antioxidant potential of ***P. grandifolia*** crude methanol and its fractionated extracts (hexane, ethyl acetate and water) have been investigated, employing three different established testing systems, such as scavenging activity on 1,1-diphenyl-2-picrylhydrazyl (DPPH) radicals, reducing power assay and β-carotene method. The total phenolic content of the ***P. grandifolia*** extracts was also assessed by the Folin-Ciocalteau’s method. The ethyl acetate extract showed significantly the highest total phenolic content, DPPH scavenging ability and antioxidant activity in β-carotene bleaching assay while the hexane extract possessed significantly strongest reducing power. The data obtained in these testing systems clearly establish the antioxidant potency of ***P. grandifolia***. As such, this is the first report on the antioxidant activities of ***P. grandifolia.***

## INTRODUCTION

Free radicals are considered as important factors in the etiology of cancer, and components with antioxidant activity have received particular attention as potential inhibitors of several cancers.[1] A number of synthetic antioxidants, such as 2- and 3-*tert*-butyl-4-methoxyphenol (i.e. butylated hydroxyanisole, BHA), 2,6-di-*tert*-butyl-4-methylphenol (i.e. butylated hydroxytoluene, BHT) and tert-butylhydroquinone (TBHQ), have been added to foodstuffs to prevent the oxidation of food, but their safety has been questioned due to toxicity issue.[2] BHA and BHT have been found to be anticarcinogenic as well as carcinogenic in experimental animals. There are reports in the literature saying that BHA appeared to have tumor-initiating and tumor-promoting action.[3] Therefore, there has been considerable interest to develop natural antioxidants from botanical sources, especially edible medicinal plants, to replace synthetic antioxidants due to the long-term safety and negative consumer perception of synthetic antioxidants.[4,5] The natural antioxidants generally function as free radical scavengers and chain breakers, complexes of pro-oxidant metal ions and quenchers of singlet-oxygen formation.[6]

*Pereskia grandifolia* Haw. (Cactaceae), commonly known as “Jarum Tujuh Bilah” in Malaysia, has been traditionally used as natural remedy in folk medicine by the locals. The leaves of *P. grandifolia* are traditionally used for the treatment of cancer, high blood pressure, diabetes and diseases associated with rheumatism and inflammation. The locals generally consume the leaves either raw or as concoction brewed from fresh leaves. The leaves of *P. grandifolia* are also used as remedy for relief of gastric pain, ulcer and for revitalizing the body.[7,8] Sahu *et al*.[9] have indicated that *P. grandifolia* was used for reduction of swellings in India, as reported by Anon.[10]

In the present study, the antioxidant potency of *P. grandifolia* crude methanol and its fractionated extracts (hexane, ethyl acetate and water) have been investigated, employing three different established *in vitro* testing systems, such as scavenging activity on 1,1-diphenyl-2-picrylhydrazyl (DPPH) radicals, reducing power assay and β-carotene method. The total phenolic content of the *P. grandifolia* extracts was also assessed by the Folin-Ciocalteau’s method. To our knowledge, there is no antioxidant study reported for *P. grandifolia*. Thus, antioxidant activity of *P. grandifolia* was evaluated as it had not been determined previously.

## MATERIALS AND METHODS

### Plant sample collection and identification

Fresh leaves of *P. grandifolia* were collected from Petaling Jaya, Selangor, Malaysia, in February 2007. The samples were identified by Professor Dr. Halijah Ibrahim of Institute of Biological Sciences, Faculty of Science, University of Malaya, Malaysia, and a voucher specimen (SN01-07) was deposited at the herbarium of the Institute of Biological Sciences, Faculty of Science, University of Malaya, Kuala Lumpur, Malaysia.

### Chemicals

Gallic acid, BHA, ascorbic acid, DPPH, potassium ferricyanide, linoleic acid, Folin-Ciocalteu’s phenol reagent and β-carotene were obtained from Sigma-Aldrich Company. Trichloroacetic acid, Tween 80, methanol, hexane and ethyl acetate were purchased from Merck Company. All other chemicals used were obtained either from from Sigma- Aldrich Company (USA) or Merck Company (Germany).

### Preparation of extracts

The extracts were prepared as described previously.[7] All the extracts (methanol, hexane, ethyl acetate and water) were kept in the dark at 4°C for not more than 1 week prior to evaluation of antioxidant activities and total phenolic content.

### Determination of total phenolic content

The total phenolic content of the extracts was measured according to the Folin-Ciocalteu method described by Cheung *et al*.[11] and Singleton *et al*.[12] The concentrations of phenolic compounds in *P. grandifolia* extracts were expressed as gallic acid equivalents (GAEs). Briefly, 0.02 ml of extract of different concentrations (4, 8, 12, 16 and 20 mg/ml) and control (methanol was used instead of extract) were mixed with 1.58 ml of distilled water. Then, 0.1 ml of Folin-Ciocalteu’s phenol reagent was added to each test tube. After 3 min, 0.3 ml of saturated sodium carbonate (Na_2_CO_3_) solution (~35%) was added to the mixture. The reaction mixtures were incubated at 40°C for 30 min. Methanol was used as blank. All assays were conducted in triplicate. The absorbance was determined at 765 nm with a spectrophotometer (Hitachi U2000). Gallic acid solutions with concentrations ranging from 25 to 1000 mg/l were used for calibration. A dose response linear regression was generated by using the gallic acid standard absorbance and the levels in the samples were expressed as gallic acid equivalents (mg of GAEs/g of extract). BHA was used as positive reference standard in the study.

### Scavenging activity on 1,1-diphenyl-2-picrylhydrazyl radicals

The scavenging activity of *P. grandifolia* extracts on DPPH radicals was measured according to the method described by Cheung *et al*.[11] with some modifications. Extracts with different concentrations and control (methanol was used instead of extract) were mixed with 0.8% of DPPH solution. The reaction mixtures were incubated at room temperature and allowed to react for 30 min in the dark. All measurements were taken in dim light. The optical density was measured at 520 nm with a spectrophotometer (Hitachi U2000). Methanol was used as blank.

The scavenging activity (%) on DPPH radical was calculated according to the following equation:

$$\mathrm{scavenging}\;\mathrm{activity}(\%)=\frac{A_{\mathrm{control}}-{\mathrm{As}}_{\mathrm{sample}}}{A_{\mathrm{control}}}\mathrm{\times 100}\%$$

where *A*_control_ is the absorbance of the control and *A*_sample_ is the absorbance of the extract/standard. All assays were conducted in triplicate. Ascorbic acid and BHA were used as positive reference standards in this study.

The scavenging ability of the extracts was expressed as EC_50_ value, which is the effective concentration at which 50% of DPPH radicals were scavenged. The EC_50_ value was obtained from the graph of scavenging activity (%) versus concentration of extracts.

### Reducing power assay

The reducing power of the prepared extracts was determined according to method described by Oyaizu.[13] Briefly, each extract in varying amounts of 5, 10, 15 and 20 mg was dissolved in 1.0 ml of methanol to which was added 2.5 ml of 0.2 M phosphate buffer (pH 6.6) and 2.5 ml of 1% (w/v) solution of potassium ferricyanide. The mixture was incubated in a water bath at 50°C for 20 min. Following this, 2.5 ml of 10% (w/v) trichloroacetic acid solution was added and the mixture was then centrifuged at 1000 rpm for 10 min. A 2.5-ml aliquot of the upper layer was combined with 2.5 ml of distilled water and 0.5 ml of a 0.1% (w/v) solution of ferric chloride. Absorbance of the reaction mixture was read spectrophotometrically (Hitachi U2000) at 700 nm. Increased absorbance of the reaction mixture indicates greater reducing power. Mean values from three independent samples were calculated for each extract. Ascorbic acid and BHA were used as positive reference standards.

### β-Carotene bleaching method

The antioxidant activity of the prepared *P. grandifolia* extracts was determined according to the β-carotene bleaching method described by Cheung *et al*.[11] A reagent mixture containing 1 ml of β-carotene solution (0.2 mg/ml in chloroform), 0.02 ml of linoleic acid and 0.2 ml of Tween 80 was pipetted into a round-bottomed flask. After removing the chloroform by using a rotary evaporator (Buchi), 50 ml of oxygenated distilled water was added to the flask. The mixture was stirred vigorously to form a liposome solution. Aliquots (5 ml) of the liposome solution were transferred to a series of test tubes containing 0.2 ml of extract with different concentrations (4–20 mg/ml). Methanol or water (instead of extract) was used as control while the blank contained all the earlier chemicals (0.02 ml of linoleic acid and 0.2 ml of Tween 80 in 50 ml of oxygenated distilled water) except β-carotene solution. The absorbance of each extract was measured immediately (*t* = 0 min) at 470 nm using a spectrophotometer (Hitachi U2000). Subsequently, the reaction mixtures were incubated at 50°C. The absorbance was measured again at time intervals of 20 min for 2 h (*t* = 120 min). All samples were assayed in triplicate. BHA was used as standard. The rate of β-carotene bleaching (R) was calculated according to the equation:

$$R\;=\;\mathrm{In}(A_{0}/A_{t})/t$$

where ln is natural logarithm, *A*_0_ is absorbance at time 0, *A*_t_ is absorbance at time *t*, and *t* is 20, 40, 60, 80, 100 or 120 min. The antioxidant activity (%) was calculated in terms of percentage inhibition relative to the control, using the equation:

$$\mathrm{antioxidant}\;\mathrm{activity}(\%)=\left(\frac{R_{\mathrm{control}}-R_{\mathrm{sample}}}{R_{\mathrm{control}}}\right)\times 100\%$$

### Statistical analysis

The antioxidant data in the present study were subjected to one-way analysis of variance (ANOVA) and the significance of the difference between the means was determined by the Duncan’s multiple range tests at 95% least significant difference (*P* < 0.05).

## RESULTS AND DISCUSSION

The differences in each antioxidant activity detection system depend on the unique characteristic of each test. Previous study shows that no single testing method is sufficient to estimate the antioxidant activity of a studied sample.[14] Thus, the combination of four methods (i.e. total phenolic content using Folin-Ciocalteau’s method, scavenging activity on DPPH radicals, reducing power assay and β-carotene method) was used in this study to evaluate the antioxidant activity of *P. grandifolia*. In this study, *P. grandifolia* was extracted with methanol and further fractionated into hexane, ethyl acetate and water extracts. As indicated by Yan *et al*.,[15] a single solvent may not be enough to identify certain extracts responsible for the activity.

### Total phenolic content

There is a recent renewed interest in phenolics as most phenolics possess strong antioxidant activity when compared to vitamins C and E *in vitro*.[16] There are reports in the literature saying that there is a highly positive relation between total phenolic content and antioxidant activity in many plant species.[17,18] The Folin-Ciocalteau’s method has become a routine assay in determining the phenolic content in a test sample. In Folin-Ciocalteau’s method, the reaction condition has been adjusted to pH ~ 10 by addition of a sodium carbonate solution. Dissociation of the phenolic proton leading to a phenolic ion is capable of reducing Folin-Ciocalteau’s reagent as shown below:[19]

Mo(VI)+e→Mo(V)

**Figure 1:**
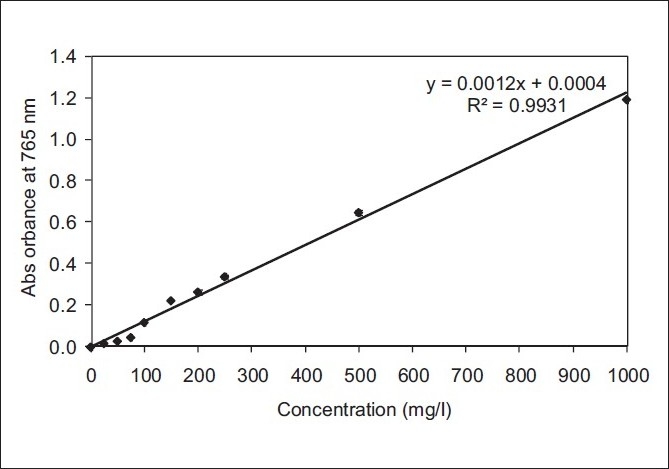
The gallic acid calibration graph

The total phenolic content in the extracts of *P. grandifolia*, determined from regression quotation of calibration curve [Figure 1], was expressed as milligrams of GAEs per gram of extract. The absorbance value of the test extract after subtraction of control (y) was translated into total phenolic content (mg/l of GAEs) using the gallic acid calibration plot with the following formula:

$$\mathrm{Total}\;\mathrm{phenolic}\;\mathrm{content}\;(\mathrm{mg1}\;\mathrm{of}\;\mathrm{GAEs})=\frac{y-0.0004}{0.0012}$$

The concentrations of total phenolics of extracts of *P. grandifolia* are shown in Table 1. The highest amount was found in the ethyl acetate extract with 45.99 mg of GAEs/g of extract, followed by the methanol extract (38.54 mg of GAEs/g of extract), water extract (35.79 mg of GAEs/g of extract) and hexane extract (19.08 mg of GAEs/g of extract), although the yield of ethyl acetate extracts was the lowest among the fractionated extracts.[7] The significantly higher (*P* < 0.05) phenolic content in the ethyl acetate extract than in the crude methanol extract was probably due to the concentration of phenolic compounds in this fractionated extract. The high phenolic content in the ethyl acetate extract might contribute toward its antioxidant activities. *P. grandifolia* contains 2,4-di-*tert*-butylphenol and α-tocopherol (also a phenolic compound) in its ethyl acetate extract.[7] It is thus reasonable to assume that the phenolic content must have been contributed by these two compounds.

**Table 1:** Concentration of total phenolics

Extracts	Concentration of total phenolics (mg of GAEs/g of extract)
Methanol	38.54 ± 0.48^a^
Hexane	19.08 ± 0.43^b^
Ethyl acetate	45.99 ± 0.30^a^
Water	35.79 ± 0.33^a^
BHA*	252.97 ± 2.81^c^

* Positive reference standard, GAEs: Gallic acid equivalents, values expressed are mean ± standard deviation of three measurements, means with different letters in the same column are significantly different (*P* < 0.05, ANOVA)

### Scavenging activity on 1,1-diphenyl-2-picrylhydrazyl radicals

The proton radical-scavenging action is known to be one of the various mechanisms for antioxidation. DPPH radical scavenging activity is determined by a colorimetric assay. This assay is sensitive, requiring only small amount of samples[20] and allows testing of both lipophilic and hydrophobic substances.[21]

DPPH is a stable free radical and accepts an electron or hydrogen radical to become a stable diamagnetic molecule.[17,18,22,23] The use of the stable DPPH radical has the advantage of being unaffected by side reactions, such as enzyme inhibition and metal chelation.[24] Scavenging of DPPH free radical determines the free radical scavenging capacity or antioxidant potential of the test sample, which shows its effectiveness, prevention, interception and repair mechanism against injury in a biological system.[25]

The scavenging activity (EC_50_ values) of extracts on DPPH radicals are shown in Table 2. Lower EC_50_ value indicates stronger ability of the extract to act as DPPH scavenger while the higher EC_50_ value indicates the lower scavenging activity of the scavengers as more scavengers were required to achieve 50% scavenging reaction. The water extract of *P. grandifolia* with EC_50_ value of >5 mg/ml was not effective in scavenging DPPH radical compared with other extracts. The ethyl acetate extract of *P. grandifolia* showed the best DPPH scavenging activity with the lowest EC_50_ 140 μg/ml, followed by the hexane (EC_50_ 285μg/ml) and methanol extract (EC_50_ 860μg/ml). This indicated that the ethyl acetate extract may well react with free radicals, terminating the chain reaction of free radicals. The stronger scavenging activity in the ethyl acetate and hexane extracts than in the crude methanol extract was probably due to the concentration of antioxidant compounds in the respective extracts.

**Table 2:** Scavenging activity (EC_50_ values) of extracts on DPPH radicals

Extracts	EC_50_ values
Methanol	860 μg/ml
Hexane	285 μg/ml
Ethyl acetate	140 μg/ml
Water	> 5 mg/ml
Ascorbic acid*	19 μg/ml
BHA*	11 μg/ml

* Positive reference standard

The results [Tables 1 and 2] show that there is a correlation between higher DPPH scavenging activity and larger amount of total phenolics in the ethyl acetate extract. This finding is supported by previous reports which showed that phenolic compounds generally correlate with antioxidant capacities measured by DPPH assay.[26,27] Thus, this indicated that phenolic compounds in the ethyl acetate extract may contribute to the DPPH scavenging activity although other antioxidants may probably be present in the extract.

### Reducing power assay

Antioxidant effect often correlates with reductive activity.[28] In the reducing power assay, the presence of antioxidants in the samples results in the reduction of the ferric cyanide complex to the ferrous form which can be monitored by measuring the formation of Pearl’s Prussian blue at 700 nm. The increased absorbance at 700 nm indicates an increase in reducing power of samples.[29] The extracts that showed comparable absorbance readings with ascorbic acid and BHA are considered to have high reducing power.

**Figure 2:**
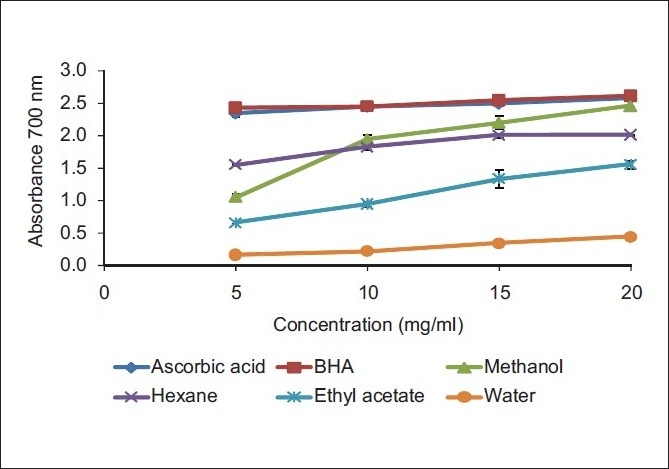
Reducing powers at various concentrations

The reducing power of *P. grandifolia* extracts and positive reference standards is shown in Table 3 and Figure 2. The reducing power of *P. grandifolia* extracts increased steadily with increasing concentrations [Figure 2] and varied significantly with different concentrations (*P* < 0.05) [Table 3]. The methanol and hexane extracts appeared to possess (*P* < 0.05) the highest significant reducing activity among the extracts [Figure 2]. The reducing powers of methanol extract were 1.056, 1.946, 2.204 and 2.460 when tested at concentrations of 5, 10, 15 and 20 mg/ml, respectively, while the reducing powers of hexane extract was 1.557 at 5 mg/ml and 2.015 at 20 mg/ml [Table 3]. The reducing power of water extract was significantly (*P* < 0.05) the lowest at 0.168, 0.227, 0.350 and 0.466 when tested at concentrations of 5, 10, 15 and 20 mg/ml, respectively. However, the reducing power of the positive reference standards (ascorbic acid and BHA) were relatively more pronounced than the tested extracts. The stronger reducing power in the hexane fractions than in the crude methanol extracts was probably due to the concentration of antioxidant compounds in the extract.

**Table 3:** Reducing powers at various concentrations

Extracts	Concentrations of extracts (mg/ml)			
	5	10	15	20
Methanol	1.056 ± 0.05^aw^	1.946 ± 0.08^bw^	2.204 ± 0.10^cw^	2.460 ± 0.00^dw^
Hexane	1.557 ± 0.02^ax^	1.827 ± 0.03^bx^	2.009 ± 0.03^cx^	2.015 ± 0.01^cx^
Ethyl acetate	0.664 ± 0.00^ay^	0.952 ± 0.04^by^	1.343 ± 0.14^cy^	1.560 ± 0.06^dy^
Water	0.168 ± 0.00^az^	0.227 ± 0.01^bz^	0.350 ± 0.01^cz^	0.466 ± 0.01^dz^
Ascorbic acid*	2.343 ± 0.05^a^	2.451 ± 0.02^b^	2.496 ± 0.02^b^	2.579 ± 0.04^c^
BHA*	2.432 ± 0.01^a^	2.458 ± 0.03^a^	2.549 ± 0.02^b^	2.616 ± 0.02^c^

* Positive reference standard, Absorbance values are expressed are mean ± standard deviation of triplicate measurements. For the same extract or standard with different concentrations, means in the same row with different letters (a–d) were significantly different (*P* < 0.05, ANOVA). For different extracts with the same concentration, means in the same column with different letters (w–z) were significantly different (*P* < 0.05, ANOVA)

### β-Carotene bleaching activity

In the β-carotene bleaching assay, the linoleic acid free radical, formed upon the abstraction of a hydrogen atom from one of its diallylic methylene groups, attacks the highly unsaturated β-carotene molecules. As a result, β-carotene molecules lose their double bonds by oxidation in this model system. In the absence of an antioxidant, the β-carotene molecule loses its chromophore and undergoes rapid discoloration, which can be monitored spectrophotometrically.[30–32]

**Figure 3:**
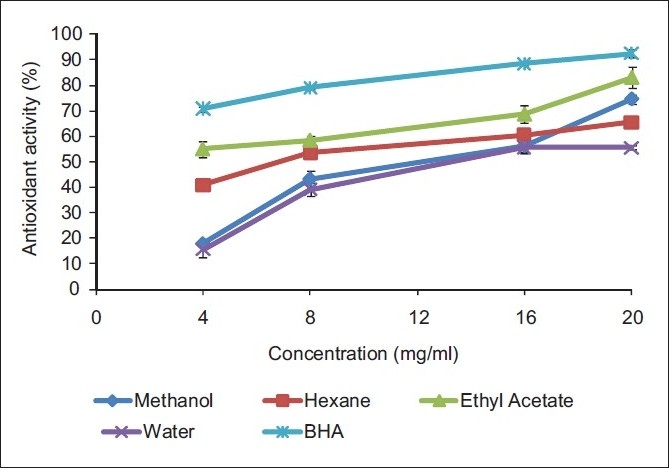
Antioxidant activity (%) measured by β-carotene bleaching method

Table 4 and Figure 3 show the antioxidant activities of the *P. grandifolia* extracts and BHA, as measured by the bleaching of β-carotene. The antioxidant activities of all the extracts gradually increased with increasing concentration of the extracts [Figure 3] and varied significantly with different concentrations (*P* < 0.05) [Table 4]. As shown in Figure 3, the water extract showed the lowest significant antioxidant activity (*P* > 0.05) while the ethyl acetate extract showed the highest significant activity at all the concentrations tested (*P* > 0.05). The ethyl acetate extracts of *P. grandifolia* exhibited 83.13% antioxidant activity at 20 mg/ml which was comparable to that of BHA standard at 20 mg/ml (92.46%) [Table 4]. The high antioxidant activity of ethyl acetate extract tested using β-carotene model may be correlated with the high phenolic content of the ethyl acetate extract [Table 1].

**Table 4:** Antioxidant activity (%) measured by β-carotene bleaching method

Extracts	Concentrations of extracts (mg/ml)			
	4	8	16	20
Methanol	17.83 ± 0.94^aw^	43.13 ± 3.58^bw^	56.21 ± 2.63^cw^	74.48 ± 1.94^dw^
Hexane	40.97 ± 0.64^aw^	53.64 ± 0.75^bw^	60.54 ± 2.08^cw^	65.51 ± 1.76^dx^
Ethyl acetate	55.04 ± 3.12^ax^	58.53 ± 1.52^ax^	68.68 ± 3.47^bw^	83.13 ± 3.99^cy^
Water	15.66 ± 2.88^ay^	39.22 ± 2.52^by^	55.68 ± 1.96^cx^	55.60 ± 0.82^cz^
BHA*	70.80 ± 1.09^a^	79.00 ± 0.52^b^	88.56 ± 0.82^c^	92.46 ± 2.52^d^

* Positive reference standard, Absorbance values are expressed are mean ± standard deviation of triplicate measurements. For the same extract or standard with different concentrations, means in the same row with different letters (a–d) were significantly different (*P* < 0.05, ANOVA). For different extracts with the same concentration, means in the same column with different letters (w–z) were significantly different (*P* < 0.05, ANOVA)

## CONCLUSION

The results obtained from the present study indicated that the ethyl acetate extract of *P. grandifolia* has the highest significant (*P* < 0.05) total phenolic content, DPPH scavenging ability and antioxidant activity (in β-carotene bleaching assay) among the extracts. This indicated that the antioxidant activity of the ethyl acetate extract was well correlated with the content of its phenolic compounds. Based on our previous study,[7] 2,4-di-tert-butylphenol, α-tocopherol and β-sitosterol were isolated and identified from the ethyl acetate extract of *P. grandifolia*. These compounds have been reported to possess antioxidant activities.[33–38] It is suggested that the total phenolic content and antioxidant activity of the ethyl acetate extract may be partly contributed by the above compounds.

In conclusion, this study suggested that *P. grandifolia* is a potential source of natural antioxidants. However, further investigations on *in vivo* antioxidant activities are highly recommended.
